# A Multiple Reaction Monitoring (MRM) Method to Detect Bcr-Abl Kinase Activity in CML Using a Peptide Biosensor

**DOI:** 10.1371/journal.pone.0056627

**Published:** 2013-02-20

**Authors:** Tzu-Yi Yang, Christie L. Eissler, Mark C. Hall, Laurie L. Parker

**Affiliations:** 1 Department of Medicinal Chemistry and Molecular Pharmacology, Purdue University, West Lafayette, Indiana, United States of America; 2 Department of Biochemistry, Purdue University, West Lafayette, Indiana, United States of America; 3 Purdue University Center for Cancer Research, Purdue University, West Lafayette, Indiana, United States of America; Stanford University, United States of America

## Abstract

The protein kinase Bcr-Abl plays a major role in the pathogenesis of chronic myelogenous leukemia (CML), and is the target of the breakthrough drug imatinib (Gleevec™). While most patients respond well to imatinib, approximately 30% never achieve remission or develop resistance within 1–5 years of starting imatinib treatment. Evidence from clinical studies suggests that achieving at least 50% inhibition of a patient’s Bcr-Abl kinase activity (relative to their level at diagnosis) is associated with improved patient outcomes, including reduced occurrence of resistance and longer maintenance of remission. Accordingly, sensitive assays for detecting Bcr-Abl kinase activity compatible with small amounts of patient material are desirable as potential companion diagnostics for imatinib. Here we report the detection of Bcr-Abl activity and inhibition by imatinib in the human CML cell line K562 using a cell-penetrating peptide biosensor and multiple reaction monitoring (MRM) on a triple quadrupole mass spectrometer. MRM enabled reproducible, selective detection of the peptide biosensor at fmol levels from aliquots of cell lysate equivalent to ∼15,000 cells. This degree of sensitivity will facilitate the miniaturization of the entire assay procedure down to cell numbers approaching 15,000, making it practical for translational applications in patient cells in which the limited amount of available patient material often presents a major challenge.

## Introduction

Kinase inhibitor drugs represent an approximately $10 billion market in the pharmaceutical industry, and this is anticipated to expand even further over the coming decade. [1] The classic example and breakthrough drug for this therapeutic strategy is Gleevec® (imatinib). Imatinib inhibits the Bcr-Abl kinase, an oncogene encoded on the Philadelphia chromosome (a translocation of chromosomes 9 and 22; tumor cells with this translocation are known as Ph+) on which the disease process of chronic myelogenous leukemia (CML) depends. Approximately 90% of CML patients achieve initial remission with imatinib, and for ∼70% of patients, that remission remains stable for a long period of time; however, a significant proportion (∼30%) either never respond or develop recurrent and/or resistant disease and experience relapse within a few years. [2], [3] Evidence from monitoring the relative inhibition of Bcr-Abl in CML patients beginning therapy suggests that failure to achieve at least 50% relative inhibition of the kinase’s activity is associated with poorer short- and long-term outcomes. [4] A similar relationship has been observed for other promising kinase inhibitor drugs. [5] Relatedly, failure of clinical trials for inhibitors targeted at other kinases due to heterogeneity of patient response (unlike the relatively homogenous response of CML patients to imatinib) is a major risk in kinase inhibitor drug development, and often arises due to a lack of pharmacodynamic assessment of the response of the drug target and downstream signaling pathways. [5], [6] The Food and Drug Administration has become increasingly involved in efforts to include companion diagnostics as a part of the drug approval process. [7] Kinase assays developed as companion diagnostics could assist with earlier decision making on the potential for a drug to be successful. This could also enable more effective selection of patients who are likely to benefit, refining the population for defining successful response in a clinical trial. [5] Additionally, the patent on Gleevec has recently expired; however, imatinib is currently first-line therapy for CML, and still holds the biggest market. [8] The still-branded inhibitors Tasigna® (nilotinib) and Sprycel® (dasatinib) are being promoted as first-line therapies for newly-diagnosed CML patients, but may not be necessary in most cases where imatinib is effective but just not reaching the relative inhibition required for the best clincial outcomes. Accordingly, stakeholders including patients, doctors and insurance companies would benefit from information on whether less costly, generic imatinib could be effective for a given individual. Pharmaceutical companies may also find methods to detect sensitivity to branded drugs useful to support the need for their branded drugs, for example in cases where imatinib is found to be ineffective regardless of dose.

A relevant companion diagnostic for kinase inhibitor pharmacodynamics needs to measure enzymatic activity, and thus substrate phosphorylation, for a targeted kinase. Protein and peptide phosphorylation by kinases has traditionally been detected using ^32^ P-radiolabeled ATP or antibody-based methods (such as ELISA and Western blot). These methods are reliable and well-characterized, but often are limited by concerns over waste generation (radiolabeled assays) or the requirement for phosphosite-specific antibodies, which are not always available at the scales necessary for clinical tests at a reasonable cost. To overcome these limitations, several approaches (e.g. microarray, bead-based and targeted mass spectrometry (MS) methods) have been described that detect kinase activity from relatively small amounts of cell lysate (down to ∼10 µg). [9]–[15] The number of Ph+, Bcr-Abl expressing CML cells per ml of blood is highly variable from patient to patient, reaching several hundreds of millions during chronic phase (during which diagnosis typically occurs) and dropping to 0.15–5×10^6^ upon hematological remission (when patients would likely benefit from monitoring for maintenance of kinase inhibition and potential for disease recurrence). Typical total protein yields from CML model cells such as K562 average approximately 50–250 µg per 10^6^ cells, so assays that can achieve reproducible signal with ∼10 µg of sample would potentially be appropriate for translational assays on patient material. However, kinase signaling is highly dependent on factors such as subcellular localization and scaffolding–therefore, assays that can measure kinase activity in intact cells are desirable for achieving the most biologically-relevant measurements of activation and inhibition. Furthermore, there is evidence from proteomic studies that downstream phosphorylation sites (such as Y207 of CrkL) may not be accurate reporters of drug sensitivity and pharmacodynamics in leukemias including CML. [16] Accordingly, exogenous sensors that report kinase activation states (rather than the phosphorylation of downstream substrates) with high sensitivity may provide useful information about individual patient response to kinase inhibitors and facilitate personalized therapeutic decisions in the clinic.

**Figure 1:**
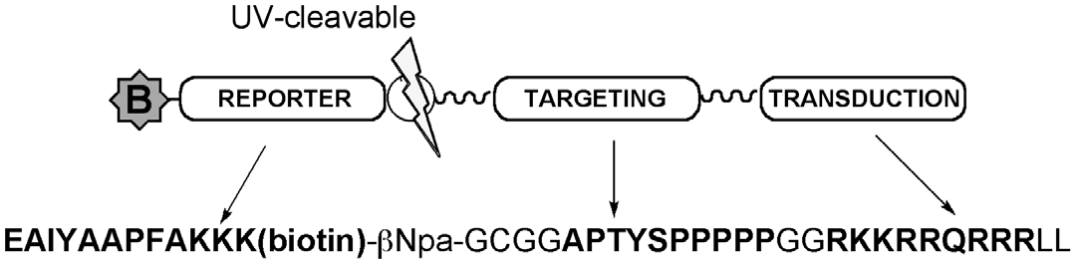
Peptide-based biosensor for Abl kinase. The ‘reporter’ sequence, called ‘Abltide,’ [28] is an efficient Abl kinase substrate. The ‘targeting’ sequence binds to the Abl kinase SH3 domain [29] (a protein-protein interaction domain important for Abl kinase regulation). The ‘transduction’ sequence is the TAT peptide from the HIV-TAT protein that helps the virus deliver its contents to host cells. Glycine linkers allow flexible spacing of active modules. A UV cleavable linker (β-Npa) allows release of the short reporter segment from the larger peptide if desired (but is not relevant to the application described here) and a biotin tag on the reporter enables detection or enrichment of the peptide via streptavidin.

Genetically-engineered Förster resonance energy transfer (FRET) protein constructs for detecting Bcr-Abl activity in intact cells have been reported, however transfecting patient cells with sensor constructs for clinical assays is very challenging and not practical for a typical clinical laboratory. We previously reported a cell-permeable peptide biosensor for Abl kinase and its application for detecting DNA damage-related Abl kinase activation in intact cells (Fig. 1). [17], [18] Because it can be applied in a relatively simple workflow (involving straightforward, reproducible incubation and lysis steps), this peptide biosensor strategy has the potential to be compatible with clinical laboratories. Our previous applications of the assay used Western blot and MALDI-TOF MS to detect peptide phosphorylation and required 2–5×10^6^ cells per experiment, which corresponds to 6–15×10^6^ cells for triplicate analyses. While these cell numbers are trivial to achieve in cultured cell lines and may be available from patient material in some cases at diagnosis, they are not optimal for application to a clinical setting in which the yield of CML cells per ml of whole blood is typically lower than those levels once treatment with imatinib is initiated. Moreover, our previous demonstrations of the assay technology were performed in model cell lines genetically engineered to overexpress constructs of Abl kinase that were not necessarily relevant to the CML disease model. Accordingly, we set out to establish an assay that would provide higher sensitivity for detecting Bcr-Abl kinase activity using the peptide biosensor and also demonstrate the method in a more clinically relevant model (the human CML cell line K562). We chose LC/MS with multiple reaction monitoring (MRM) as an analysis strategy based on its excellent sensitivity and signal to noise. Lower detection limits for MRM can reach the fmol-amol range depending on the specific analytes and instrumentation involved. We and others[9], [19]–[21] have successfully detected and quantified peptides and their phosphorylated derivatives using this technique. Here we describe the development of the MRM method and demonstrate that the biosensor/MRM strategy can be employed to detect Bcr-Abl activity and inhibition in CML cells as a model for monitoring drug sensitivity in human leukemia.

## Materials and Supplies

### Peptide Synthesis

Fmoc-protected amino acid monomers were purchased from Peptides International (Louisville, KY, USA). Fmoc–biotinylated lysine was obtained from Akaal Organics (Long Beach, CA, USA). The photocleavable residue (3-(2-nitrophenyl)-3-aminopropionic acid) was obtained from Lancaster Synthesis and Fmoc-protected by J. Thomas Ippoliti’s lab at the University of St. Thomas (St. Paul, MN). Other reagents were purchased from Sigma-Aldrich if not specified.

### Cell Culture

K562 cells were obtained from ATCC (Rockville, MD). Cells were routinely maintained in RPMI-1640 supplemented with 10% fetal bovine serum, 1% penicillin/streptomycin, and 1% L-glutamine in a 5% CO2 humidified environment at 37°C.

### Experimental Procedures

#### Peptide synthesis, purification and characterization

Peptides were synthesized via solid phase Fmoc chemistry on 50 µmol CLEAR-Amide resin using a Prelude Peptide Synthesizer (Protein Technologies, Tucson, AZ, USA) with a double coupling cycle (22 min deprotection with 20% piperidine in DMF, 6×1 ml DMF wash, 2×10 min coupling with 100 mM amino acid, 90 mM HCTU/0.4 M NMM in DMF, 3×1 mL DMF wash). After synthesis, peptides were deprotected and cleaved using TFA/Water/EDT/TIS 94.5%/2.5%/2.5%/1%. After cleavage, peptides were purified to at least 90% purity using an Agilent Technologies 1200 Series HPLC system (Santa Clara, CA, USA) with a C18 reverse phase column (1×25 cm). Characterization of peptide identity and purity was performed by LC/MS (Accela/LTQ, Thermo Finnigan) with a Hypersil GOLD column (2.1×50 mm) and MALDI-TOF/TOF MS (Voyager 4800, Applied Biosystems, Foster City, CA, USA). Peptides were lyophilized and stored at −20°C before use.

#### Cell-based biosensor assay

Three independent replicate experiments were performed for the time course with Western blot detection. Three side-by-side replicate experiments with just one time point (5 min) were performed for the MRM analysis. K562 cells were cultured to log phase growth and seeded to 5×10^6^ cells/ml in a six-well plate (3 mL per well). When necessary, cells were pre-incubated with imatinib (10 µM) for 1 h at 37°C followed by incubation with three treatments: 25 µM peptide (dissolved in PBS), 25 µM peptide +10 µM imatinib, and 25 µM peptide +1 µM pervanadate (prepared by reacting a solution of sodium orthovanadate with H_2_O_2_, followed by heating at 95°C to degrade excess H_2_O_2_). At the indicated time points (5, 30, 60 min), aliquots (1 mL) were collected and centrifuged (2200 rcf, 1 min, 4°C) to remove excess media. To collect any remaining cells in the wells from the final aliquot, all wells were washed with phosphate buffered saline (PBS, 400 µl) and these washes were combined with the collected cells. Cells were suspended in PBS (1 ml) to wash away excess peptide, centrifuged again (2200 rcf, 1 min, 4°C), and lysed using Phosphosafe Extraction Reagent (Novagen) supplemented with EDTA and protease inhibitor cocktail (Roche). Cells were immediately flash-frozen in liquid nitrogen, thawed on ice for 15 min, vortex mixed, and centrifuged to clarify (16,000 rcf, 15 min, 4°C). The supernatant was collected, measured for total protein concentration using the BCA assay (ThermoFisher Pierce, Rockford, IL), flash frozen again and stored at −80°C until use.

#### Enrichment and MALDI-TOF/TOF analysis

Biosensor peptide from samples generated as described above (in the Cell-based biosensor assay section) was captured using streptavidin-coated MagneSpheres (Promega Corporation, Madison, WI). The beads (20 µl) were prepared by washing with 0.1% Octyl-β-glucoside/PBS (3×150 µl). K562 cell lysates (200 µg total protein) were incubated with the beads on a shaker (600 rpm, 60 min). Beads were captured using a MagnaBot 96-well magnetic capture device (Promega) and washed with 0.1% Octyl-β-glucoside/PBS (3×150 µl) and deionized water (3×150 µl). Peptide was eluted using 15 µL sample buffer (ACN/H2O/TFA, 50%/50%/0.1%). 0.5 µL from each sample was co-spotted with α-cyano-4-hydroxycinnamic acid (10% w/v) containing ammonium dihydrogen phosphate (5 mg/ml), [22] dried and analyzed on a 4800 MALDI-TOF/TOF Analyzer (ABSciex). Selected ions were analyzed by MS/MS (specifying the parent ion mass for selection and CID) and sequenced de novo.

#### Western blot analysis

Samples of equal protein content (100 µg/lane) were diluted into Laemmli buffer and subjected to SDS-PAGE. Proteins were transferred to nitrocellulose membrane and analyzed by Western blotting. Membranes were split at the 15 kD mark and blocked in 3% milk in TBS-T overnight at 4°C, followed by blotting with the indicated antibodies in 3% milk/TBS-T. The bottom membrane was blotted with: DyLight-649 labeled Streptavidin (1∶1000, ThermoFisher Pierce) to detect total biosensor; 4G10 α-phosphotyrosine antibody to detect the phosphorylated biosensor. Upper section of the membrane was blotted with: α-phospho-Abl (Y245) (1∶1000, Cell Signaling), α-phospho-STAT5 (Y694) (1∶5000, Abcam) and α-phospho-CrkL (Y207) (1∶1000, Abcam) to detect phosphorylation of endogenous sites in the Bcr-Abl signaling pathway. α-β-tubulin (1∶100,000, Millipore) was used as a loading control. Blots were incubated with IR-dye-labeled secondary antibodies (Rockland Immunochemical) in 3% milk/TBS-T (1∶10,000). Signals of immunoblots were visualized using the Odyssey system (LiCOR Biosciences, Lincoln, NE), quantified using densitometry with Quantity One (Bio-Rad), and analyzed with GraphPad Prism software.

#### Sample preparation and digestion

Aliquots (18 µg each) of cell lysate samples were processed to separate proteins from lipids. [23] Chloroform/methanol/water (2∶2:1.8 v/v/v) was added to the samples and the mixture vortex mixed, followed by centrifugation (5 min at 3000 rpm) to separate the chloroform and aqueous layers. The aqueous layer was retained and extracted again in the same manner, after which the chloroform layers containing lipids were discarded. The extracted protein in the aqueous layer was then precipitated with cold acetone prior to the digestion protocol, in which the samples were taken up in ammonium bicarbonate buffer (50 mM) containing 0.1% (w/v) RapiGest SF (Waters Corporation, Milford, MA) to give protein concentrations of 1 µg/µl. Dithiothreitol (DTT, 10 mM) was added (1∶1 per volume, for a final concentration of 5 mM) and the sample incubated at 60°C for 30 min to denature the proteins. After cooling to room temperature, samples were incubated in the dark with iodoacetamide (final concentration 15 mM from 55 mM stock) for 30 min. Trypsin was added (0.5 µg) and the samples incubated at 37°C for 18 h. Following digestion, samples were treated with TFA (10% stock to give final concentration 0.2% w/v) at 37°C for 30 min. Samples were diluted with 0.01% TFA to 0.5 µg/µl, centrifuged to clarify, and the supernatant injected directly onto the triple quadrupole LC/MS system (described below) for analysis (2 µl per sample, for a total protein loading of ∼1 µg). For calibration standards, Abl biosensor peptide and synthetic reporter segment were added into lysate at appropriate concentrations to result in 5–250 fmol per 2 µl injection, then digested and prepared as described above.

#### LC-MS/MS analysis

Tryptic peptides were separated on a nano-LC/MS system which included Agilent 1100 Series capillary and nano flow pumps, micro-well plate sampler with thermostat, and Chip Cube MS interface on the Agilent 6410 Triple Quadrupole mass spectrometer (Agilent Technologies, Santa Clara, CA). The peptides were loaded at 3 µl/min on an Agilent chip containing a 40 nl enrichment column packed with Zorbax 300SB-C18 5 µm material. The enrichment column was switched into the nano flow path after 5 min, and peptides were separated with an analytical column (0.75 µm×150 mm) packed with C18 reverse phase ZORBAX 300SB-C18 5 µm material at a flow rate of 0.3 µl/min. The chip is coupled to the electrospray ionization (ESI) source of the triple quadrupole mass spectrometer. The peptides were eluted from the column using a linear gradient of increasing acetonitrile. For the first 5 min, the column was equilibrated with 5% acetonitrile/95% water/0.1% formic acid (mobile phase A) followed by a linear gradient of 5%–15% B (100% acetonitrile/0.1% formic acid) in 10 min, 15–22% B in 30 min, and 22–100% B in 35 min. The column was washed with 100% B and then equilibrated with A before the next sample was injected. Blank injections were run between samples to avoid carryover.

Product ion scans were run on the triple quadrupole instrument and analyzed using Skyline software [24] (MacCoss Labs, WA) to develop a method to monitor transitions from each peptide of interest. Quadrupole 1 and 3 were run at unit resolution with a minimum dwell time of 30 msec. Using this method, peptides of interest were analyzed by multiple reaction monitoring mass spectrometry (MRM-MS). Standard peptides were synthesized and diluted into stock solutions in deionized water (using dry weight as measured by analytical microbalance) and concentration curves (in fmol) were used for quantitation of the peptides in the cell lysate samples.

## Results and Discussion

### Characterization of Biosensor Uptake and Phosphorylation

**Figure 2:**
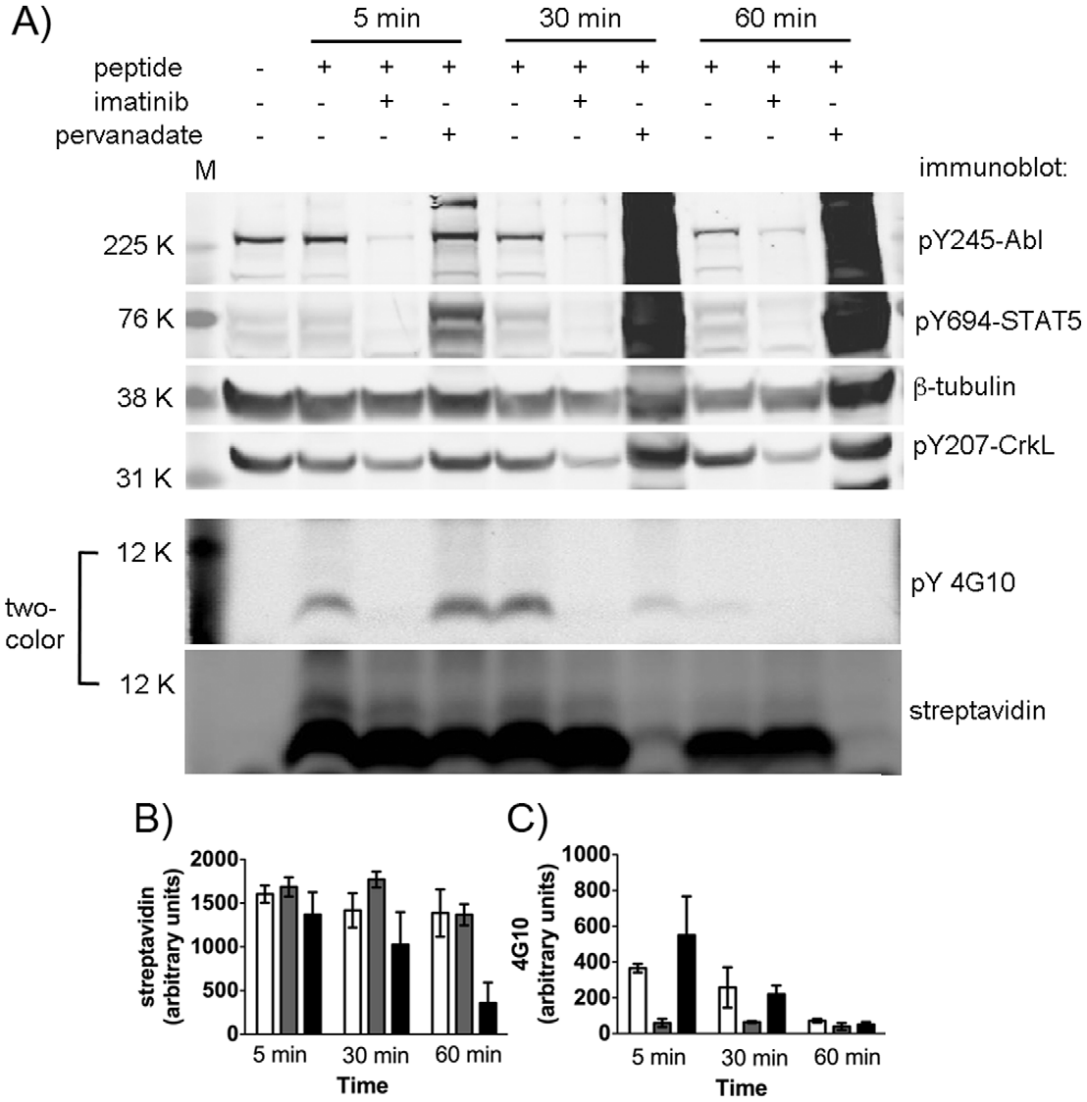
Bcr-Abl activity detection using Abl substrate peptide in the human CML cell line K562. Bcr-Abl activity was detected using the biosensor peptide in K562 cells at 5, 30 and 60 min (N = 3). Peptide and phosphopeptide were detected below the 15 kD molecular weight marker by overlaid signal from IR-dye-labeled streptavidin (showing biotinylated peptide) and Western blot for antiphosphotyrosine (4G10). Phosphorylated endogenous proteins Abl, STAT5 and CrkL, as well as β-tubulin as a loading control, were detected as described in the Materials and Methods. Additional replicate blots shown in the supporting information. Quantification of total peptide (streptavidin bands) and phosphopeptide (4G10 bands) via raw data from integration of fluorescence intensity at the MW observed for the biosensor (without background subtraction) is shown (B) to illustrate degradation of the peptide in the presence of phosphatase inhibitor, which may have contributed to the decrease in phosphopeptide signal over time (C). White = peptide alone, grey = inhibition by Gleevec (imatinib/IM), black = phosphatase inhibitor sodium pervanadate (PV). Error bars represent SEM from three independent experiments.

We based the peptide biosensor assay for Bcr-Abl/Abl kinase activity on our previously reported methodology, [17], [18] applied here to the disease-relevant, patient-derived CML model cell line K562. We first characterized the uptake and phosphorylation of the biosensor peptide in these cells using Western blot. To confirm that intracellular Abl signaling was not disrupted in our system, we probed cell lysates to examine the phosphorylation status of Bcr-Abl’s autophosphorylation site and the endogenous Abl substrates STAT5 and CrkL. Cells were cultured to log phase growth then treated with the peptide EAIYAAPFAKKK_(γ-biotin)_G-βNpa-GCGGAPTYSPPPPPGGRKKRRQRRRLL in the presence or absence of either imatinib or the phosphatase inhibitor pervanadate for 5, 30 or 60 minutes. Then cells were harvested and lysed in detergent buffer containing EDTA (to quench and prevent post-lysis kinase activity), phosphatase inhibitors (to inhibit post-lysis dephosphorylation of the biosensor) and protease inhibitors (to inhibit post-lysis degradation) before being flash frozen in liquid nitrogen. Lysates were processed as described in the Experimental Procedures, separated by SDS-PAGE and analyzed by Western blot using a two-color LiCOR scanner for quantitative detection of IR-dye labeled secondary antibodies and streptavidin (to measure total peptide signal via the biotinylated residue). As shown in Fig. 2 and blots from additional replicates provided in the supporting information, the peptide was readily taken up into K562 cells and phosphorylated, and the presence of EDTA (quenching kinase activity) and phosphatase inhibitors (preventing dephosphorylation of the peptide) in the lysis buffer indicated that the observed phosphorylation of the biosensor was occurring in the cell before lysis. As expected, phosphorylation was inhibited in the presence of imatinib and stabilized in the presence of pervanadate. In the presence of pervanadate, observed peptide levels decreased over time (as shown in Fig. 2B and 2C, plotted from raw integration of band intensities with no background subtraction). Since the lysis buffer contained a cocktail of protease inhibitors, it was unlikely that this degradation was occurring post-lysis. Consistent with previous reports, [25] we found that this was due to degradation of the peptide once it enters the intracellular environment.

**Figure 3:**
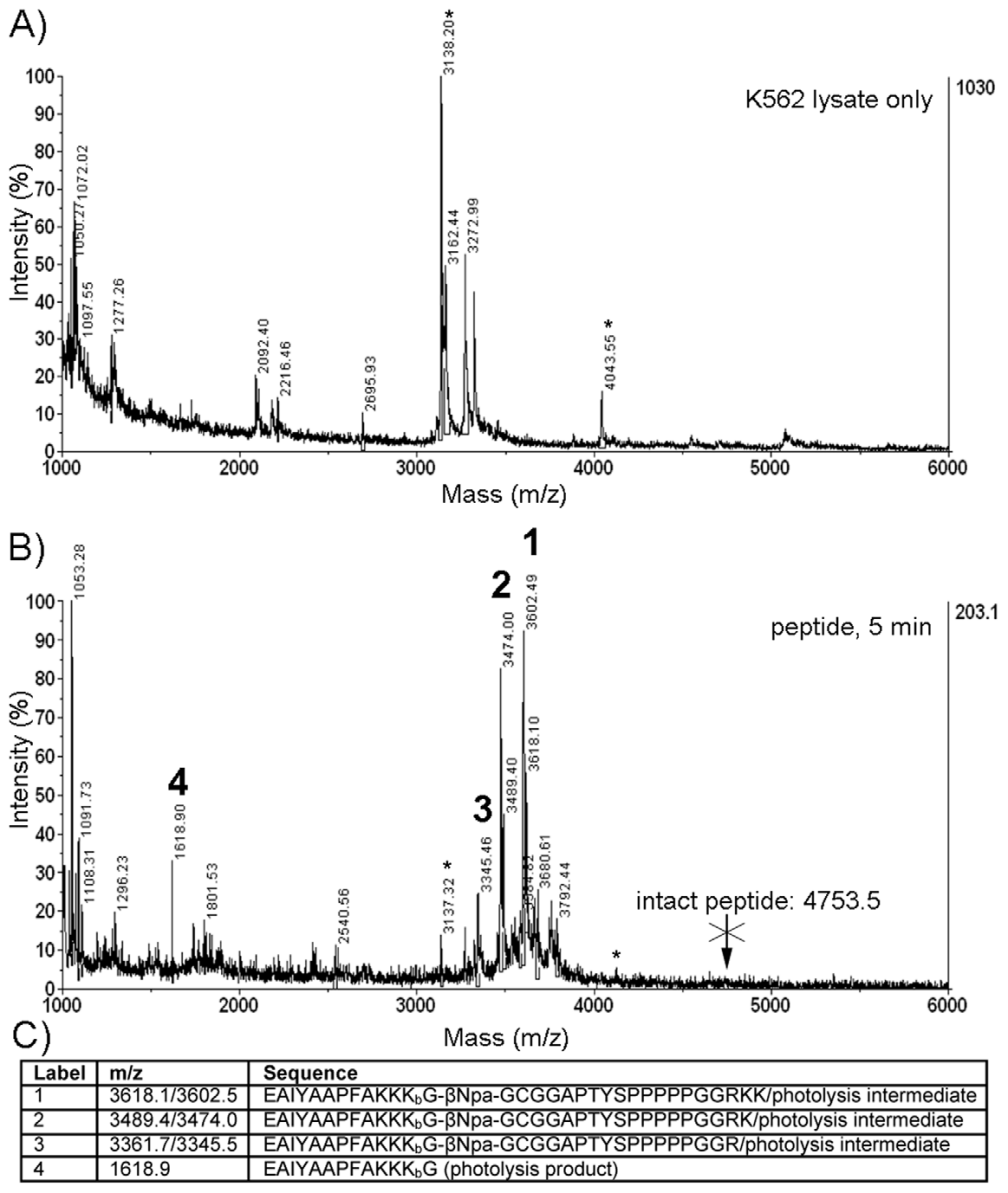
Degradation of the Abl biosensor in K562 cells. After just 5 min of incubation with cells and ∼2–3 min sample handling time, no intact peptide biosensor (Mw: 4753.5) remains. Detected ions suggest that the biosensor peptide underwent C-terminal degradation in K562 cells, leaving the N-terminal “reporter” region unperturbed. Representative degradation products are listed in the sequence table (C) and highlighted in the MALDI-TOF spectrum (B). *M/z* values listed in the table refer to each degradation product and its photocleavable linker photolysis-related derivative. Derivatives exhibiting net losses of 16 and 34 amu were also observed in the MALDI-TOF (but not ESI) MS spectra for the intact peptide. These were likely due to laser-induced fragmentation of the nitro group on the photocleavable linker as described in the supporting information; MALDI-TOF and ESI data for the intact peptide are also provided in the supporting information. A mock enrichment with K562 cell lysate alone is shown for comparison (A). MS/MS analysis of a representative degradation product (1) is shown in the supporting information.

To test this, we exploited the biotin affinity tag to capture peptide from lysate generated after 5 min incubation and analyzed it by MALDI-TOF/TOF mass spectrometry. Within just 5 min of exposure to cells (as well as approximately 2–3 min additional processing time for cell harvesting), essentially no intact peptide was observed, even for samples treated with peptide alone (Fig. 3). Several fragments were detected (along with photo-induced ion chemistry intermediates arising from the UV laser ionization inherent in MALDI-TOF analysis, also observed with the intact peptide as further discussed in the supporting information) and identified by MS/MS analysis to arise from C-terminal degradation. In particular, the cell permeability tag, TAT, was almost completely removed from the C-terminus. However, the N-terminal “reporter” sequence (which contains the phosphorylation site for Bcr-Abl, see Fig. 1) was still intact–that is, no corresponding N-terminally truncated fragments were observed. Accordingly, the bands detected by streptavidin/4G10 in Fig. 2 likely represent these degradation products. Peptide in lysates was enriched using streptavidin-coated magnetic nanoparticles through the peptide’s biotinylated reporter segment. From this workflow, phosphorylated and unphosphorylated peptides could be detected by MALDI-TOF in linear positive and negative mode, however reproducibility and signal to noise were poor (example spectra provided in the supporting information)–a problem we had not previously encountered when working with engineered cell lines. [18] Accordingly, a more sensitive detection strategy was needed to robustly quantify the degree of biosensor peptide phosphorylation by Bcr-Abl. In future work, it may be possible to improve sensitivity and reduce ambiguity by eliminating the photocleavable linker and stabilizing the peptide biosensor via the incorporation of residues resistant to proteolysis; [25], [26] however, our first goal was to establish an optimized detection technology.

### Development of the MRM Method for Quantitative Analysis

**Figure 4:**
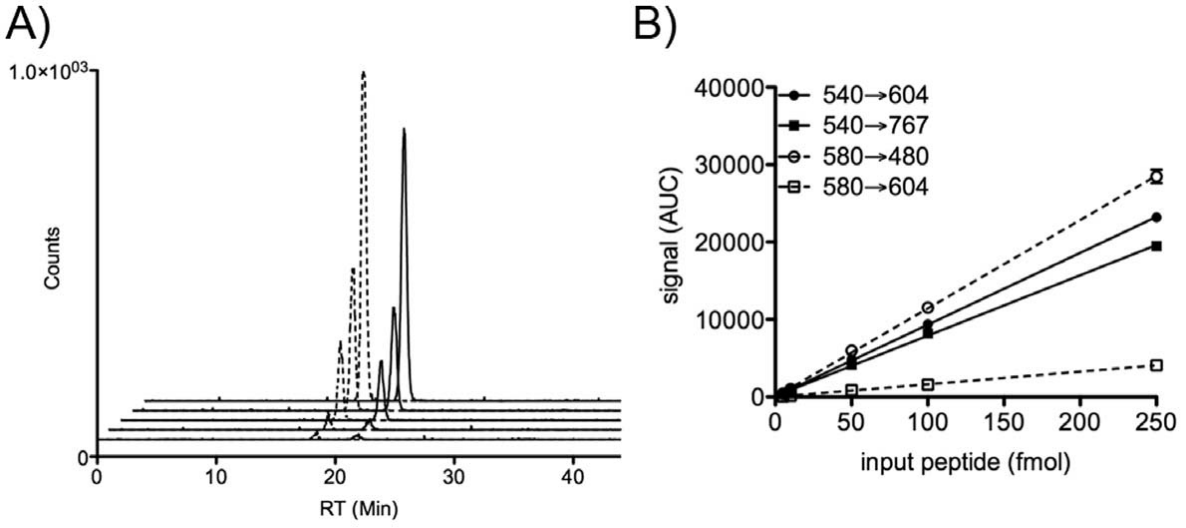
Calibration curve for detecting unphosphorylated and phosphorylated peptide using MRM. Synthetic versions of each peptide were added into trypsin-digested K562 cell lysate and analyzed by MRM. Extracted ion chromatograms for each were generated (A), integrated and plotted against input peptide to establish the calibration curve (B). Solid lines represent the 540604 transition from the unphosphorylated peptide EAIYAAPFAK; dashed lines represent the 580480 transition from the phosphorylated derivative EAIpYAAPFAK. Error bars (which are too small to be visible on the graph) represent standard deviation (SD) of two replicate analyses.

We pursued MRM as a strategy to achieve better sensitivity in the context of trypsin-digested whole cell lysate from cells incubated with the biosensor peptide. A key advantage of MRM is the ability to detect and reproducibly quantify targeted peptides and phosphopeptides [19], [20] with exquisite selectivity and sensitivity. A further advantage for the peptide biosensor assay was that the tryptic fragment arising from the “reporter” module sequence is unnatural, and thus not subject to confounding background from the native proteins in the cell lysate. Two transitions for each peptide were included in the development of the MRM method, to increase confidence in peptide identity. A calibration curve was established for quantitation of the MRM signal from the tryptic fragments of the biosensor peptide and its synthetically phosphorylated derivative, which were added 1∶1 at various concentrations into trypsin-digested K562 lysate (1 µg/µl). Signals for both the modified and unmodified form of the Abl biosensor peptide were robust and linear between 5 and 250 fmol (Fig. 4). Analytical coefficients of variation (CV) were between 1–26% (depending on the transition). One transition from each peptide was chosen for quantitative analysis based on its signal to noise and CV across the calibration range (Table 1). Based on the ratios of these transitions, the ratio of signals for the phosphorylated and unphosphorylated peptides was approximately 1∶1, with a CV of 4.3% across the entire concentration range, giving us confidence in the analytical reproducibility for quantifying the percent phosphopeptide in a sample.

**Table 1:** Transitions used to detect the Abl biosensor via MRM.

Parent ion	EAIYAAPFAK				Parent ion	EAIpYAAPFAK			
(M+2H^+2^540.8	CID (V)	LOD (fmol)	LOQ (fmol)	Average CV (mean±SD) acrossconcentration range	(M+2H)^+2^ 580.9	CID (V)	LOD (fmol)	LOQ (fmol)	Average CV (mean±SD) across concentration range
**540→604 (y_6_)** *	120.0	0.5	0.5	7±4%	**580→480 ([y_8_]^+2^)** *	80.0	0.5	5	4±4%
**540→767 (y_7_)**	120.0	0.5	1	7±10%	**580→604 (y_6_)**	80.0	5	<10	3±2%

Lower limit of detection (LOD) represents 3 SD from background (cell lysate tryptic digest alone). Lower limit of quantification (LOQ) represents 10 SD from background. Reproducibility for detecting each transition from two technical replicates across the calibrated range (5–250 fmol) is reported as the average of the CV for each transition at each concentration.

* Transitions chosen for quantitative analysis.

### Analysis of Biosensor Phosphorylation

**Figure 5:**
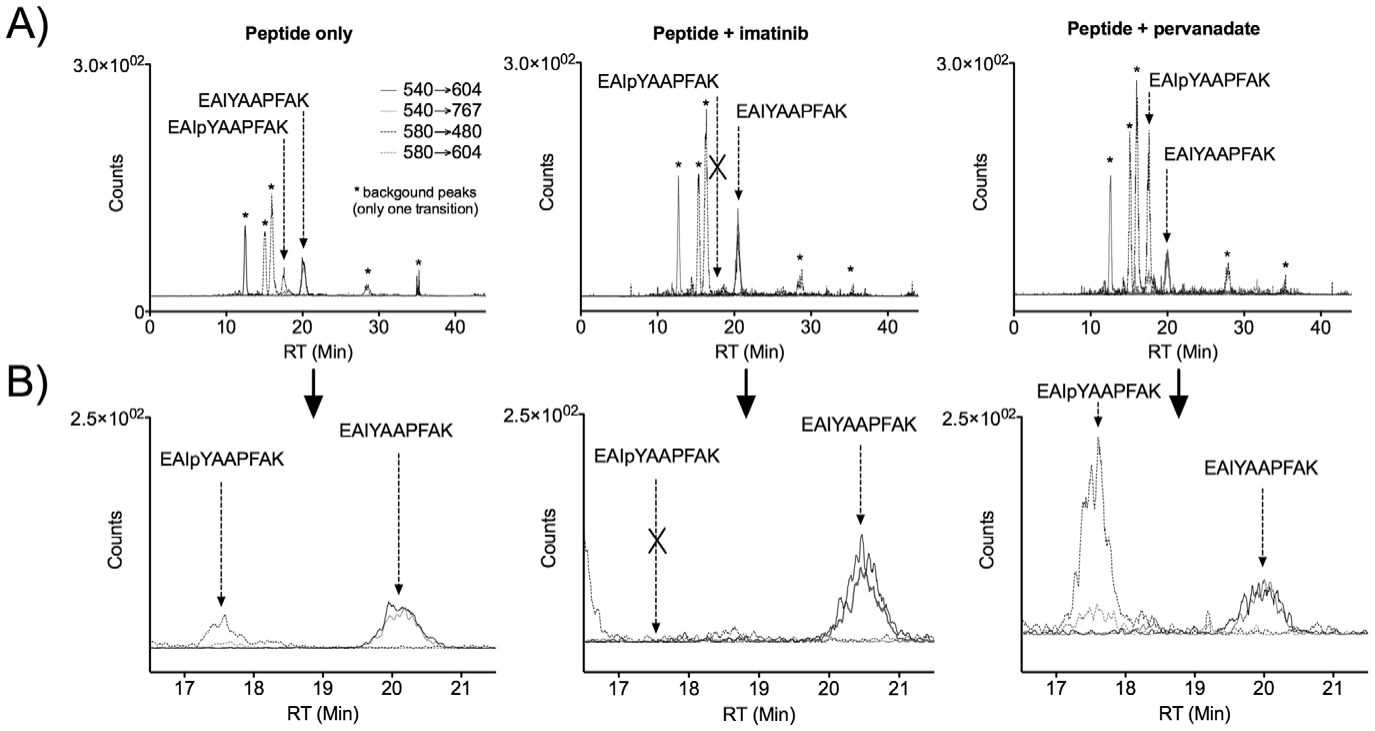
Representative extracted transition chromatograms for each treatment condition. Full chromatograms are shown in row (A). Expanded chromatograms for the region in which EAIYAAPFAK and EAIpYAAPFAK are expected to elute (based on the calibration curve) are shown in row (B), and show that the relevant peaks had signal for both transitions from each analyte.

**Figure 6:**
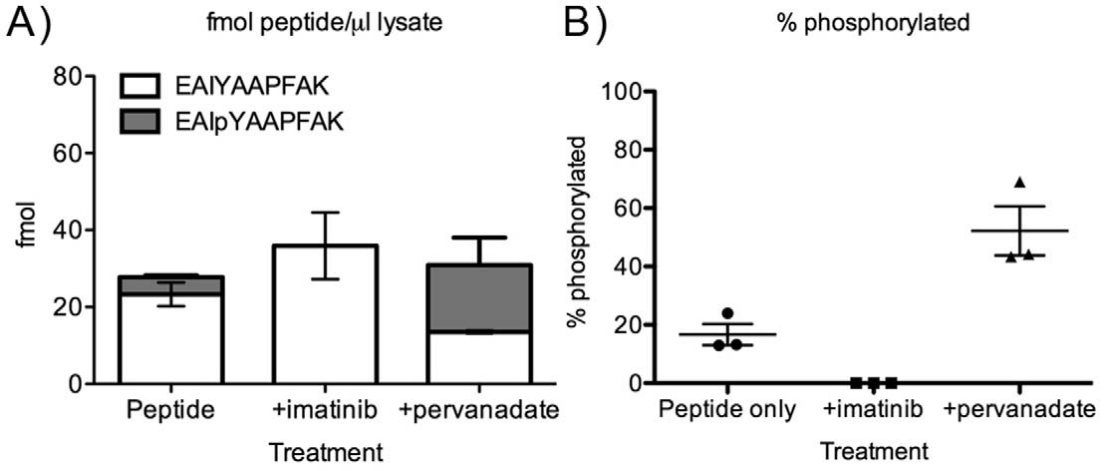
Quantification of peptide amounts and percent phosphorylation. Total peptide detected is represented by each full bar in (A), with the white portion representing the fraction of phosphopeptide and the gray fraction representing the fraction of unphosphorylated peptide. % phosphorylation is shown in (B). Error bars in both represent SEM from three replicate biological experiments.

To analyze biosensor phosphorylation, K562 cells were incubated with the biosensor for 5 minutes either alone or in the presence of Bcr-Abl inhibitor (imatinib) or phosphatase inhibitor (pervanadate) as described above. 1 µg of each cell lysate was analyzed using the MRM method described above. MRM signal data were extracted as chromatograms and the substrate peptide and its phosphorylated derivative were identified by the presence of both transitions in their respective peaks at the retention time expected for these analytes from the calibration curve analyses. Some background peaks were observed in each extracted chromatogram (Fig. 5A), however none of these exhibited signal for both transitions and the expected retention time. Because the intensities of the total ion chromatograms (TICs) for each analysis were not completely uniform, a characteristic, invariable peak in the TIC was integrated and used to calculate a correction factor for each MRM chromatogram. After this correction was applied, the peaks specific to the unphosphorylated and phosphorylated peptides were integrated and interpolated to determine the amount of unphosphorylated and phosphorylated peptides in each sample. As expected, both unphosphorylated and phosphorylated peptides were detected in the samples treated with peptide alone (Figs. 5B and 6). No phosphorylated peptide was detected in the samples pre-treated with imatinib, and higher levels (relative to peptide alone) of phosphorylated peptide were detected in the samples treated with phosphatase inhibitor. Differences between % phosphorylation observed in the peptide only and peptide+pervanadate samples were statistically significant (p<0.05, one-way ANOVA with Tukey post-test), as well as being significantly different compared to the absence of phosphopeptide seen in the peptide+imatinib samples (p = 0.044 and 0.025, respectively, one sample t-test).

All peptides were detected at levels above both their LOD and LOQ (Table 2). The fmol-scale levels of peptide detected here may represent the entirety of material taken up into cells and isolated by lysis, or it may represent the remaining reporter segment present after some degree of degradation. MALDI-TOF analysis of degradation indicated that N-terminal degradation did not appear to be taking place, however it is still possible that the fragments were just not observable. Future work to better understand biosensor stability will address this. Nonetheless, MRM-based detection of the N-terminal tryptic fragment was for the most part reproducible: CVs for analyte quantification were acceptable (20% for unphosphorylated and 23% for phosphorylated species) for the samples treated with peptide alone. CVs for the imatinib and pervanadate treated samples were somewhat higher (∼42% for total peptide detection, unphosphorylated plus phosphorylated, from each) and considerably higher (71%) for the amount of phosphopeptide detected in the pervanadate treated samples. Comparing these results to the Western blot detection described in Fig. 2, the CVs for streptavidin band intensities from the experiment described in Fig. 2 were lower for the peptide only and imatinib treated samples (11% each) but comparable (32%) for the pervanadate treated sample. CVs for the 4G10 signals of the peptide only (12%) and pervanadate (68%) samples were more similar to the CVs from MRM. For imatinib treated samples, 4G10 Western blot CVs were much higher due to the background intensity which was not a factor in the MRM analysis. While these two experiments and methods cannot necessarily be directly compared (for example, the analytical variabilities may be different between the two techniques, and the ratio of 4G10/streptavidin signal is uncalibrated and thus cannot give a % phosphopeptide), this at least shows that the results from MRM analysis are correlated with those observed using the traditional Western blot analysis. Based on the excellent analytical CVs obtained from the MRM calibration curve experiments, the higher CVs observed for the imatinib and pervanadate treated samples most likely reflect biological or sample processing and handling variability (e.g. peptide uptake, the enzymatic reaction taking place, level of phosphatase inhibition, and small differences in e.g. lysis and/or handling) rather than analytical variability. When self-normalized to represent the % phosphopeptide compared to total (Fig. 6B), CVs were within an acceptable range (20–40%) for all samples, given the biological variability involved in a cell-based enzyme activity assay. [27].

**Table 2:** Amounts and CVs for peptide and phosphopeptide detected from live cell Bcr-Abl assay samples.

	Peptide only				Peptide+imatinib				Peptide+pervanadate			
	Unphospho (fmol)	Phospho (fmol)	Total (fmol)	% phospho	Unphospho(fmol)	Phospho(fmol)	Total (fmol)	% phospho	Unphospho(fmol)	Phospho(fmol)	Total (fmol)	% phospho
**Amount**	23±5	4±0.9	28±5	17±6	36±15	ND	N/A	0	14±0.6	17±12	31±13	52±16
**CV**	23%	20%	16%	38%	42%	N/A	N/A	N/A	5%	71%	42%	28%

The amounts of analyte detected (±SD) per µg of digested sample are given in fmol. Values represent the mean of three replicate biological experiments.

Taken together, these results demonstrate that we can accurately and reproducibly detect Bcr-Abl biosensor peptide phosphorylation and inhibition in an intracellular assay. Using K562 cells as a human CML model system, we showed substantial improvements in the lower detection limits for the assay read-out compared to our previous detection strategies. The amount of total sample analyzed (1 µg) is equivalent to approximately 15,000 cells, indicating that we can achieve several orders of magnitude improvement in sensitivity compared to Western blot or MALDI-TOF detection. [17], [18] While the total number of cells processed as demonstrated in this manuscript was >250,000, this level of sensitivity and technical reproducibility for the detection method should enable future miniaturization of the assay procedure and application to clinical material.

### Concluding Remarks

In this method development work, we demonstrated a peptide biosensor-based assay for monitoring Bcr-Abl kinase activity and inhibition in intact, live cells using MRM detection with femtomole sensitivity and good reproducibility. SRM and MRM have long been established as clinical tools for monitoring small molecule metabolites, and are becoming more and more popular for analysis of protein biomarkers from blood samples. Peripheral blood mononuclear cell (PBMC) and serum samples are routinely prepared by pathology laboratories and can either be sent to contract facilities for MRM/SRM analysis or even analyzed in-house, since many hospital labs now have the necessary instrumentation and expertise. Therefore, it should be possible to establish MRM-based biosensor assays as companion diagnostics for kinase inhibitor therapy. Since mass spectrometry is also capable of detecting many analytes simultaneously, expansion of the suite of peptide biosensors to additional kinase targets for multiplexed analysis in CML and other leukemias may allow this strategy to be used in the future to analyze signaling profiles and drug sensitivity for individual patients, enabling personalized assessment of the therapeutic options from available kinase inhibitors.

## Supporting Information

Figures S1 A compiled supporting information document containing additional data is available in PDF format.
(PDF)

Table S1 Raw EIC data from MRM analyses is available as Table S1.
(ZIP)

Table S2 A table of calculations for all MRM experiments is available as Table S2.
(XLSX)
