# Supplementary material for: A Multiple Reaction Monitoring (MRM) Method to Detect Bcr-Abl Kinase Activity in CML Using a Peptide Biosensor
Source: PLoS One. 2013 Feb 20;8(2):e56627. doi: 10.1371/journal.pone.0056627 (PMC3577862; doi:10.1371/journal.pone.0056627)
Supplement: Figures S1 — A compiled supporting information document containing additional data is available in PDF format. (PDF) [file pone.0056627.s001.pdf]

Supporting information for "A multiple reaction monitoring (MRM) method to detect Bcr-Abl kinase activity in CML using a peptide biosensor," Yang et al.

Figure S1. LC/MS characterization of Abl biosensor peptide. Used in assays and also spiked into K562 lysate for digestion and use as MRM calibration standard for EAIYAAPFAK fragment.

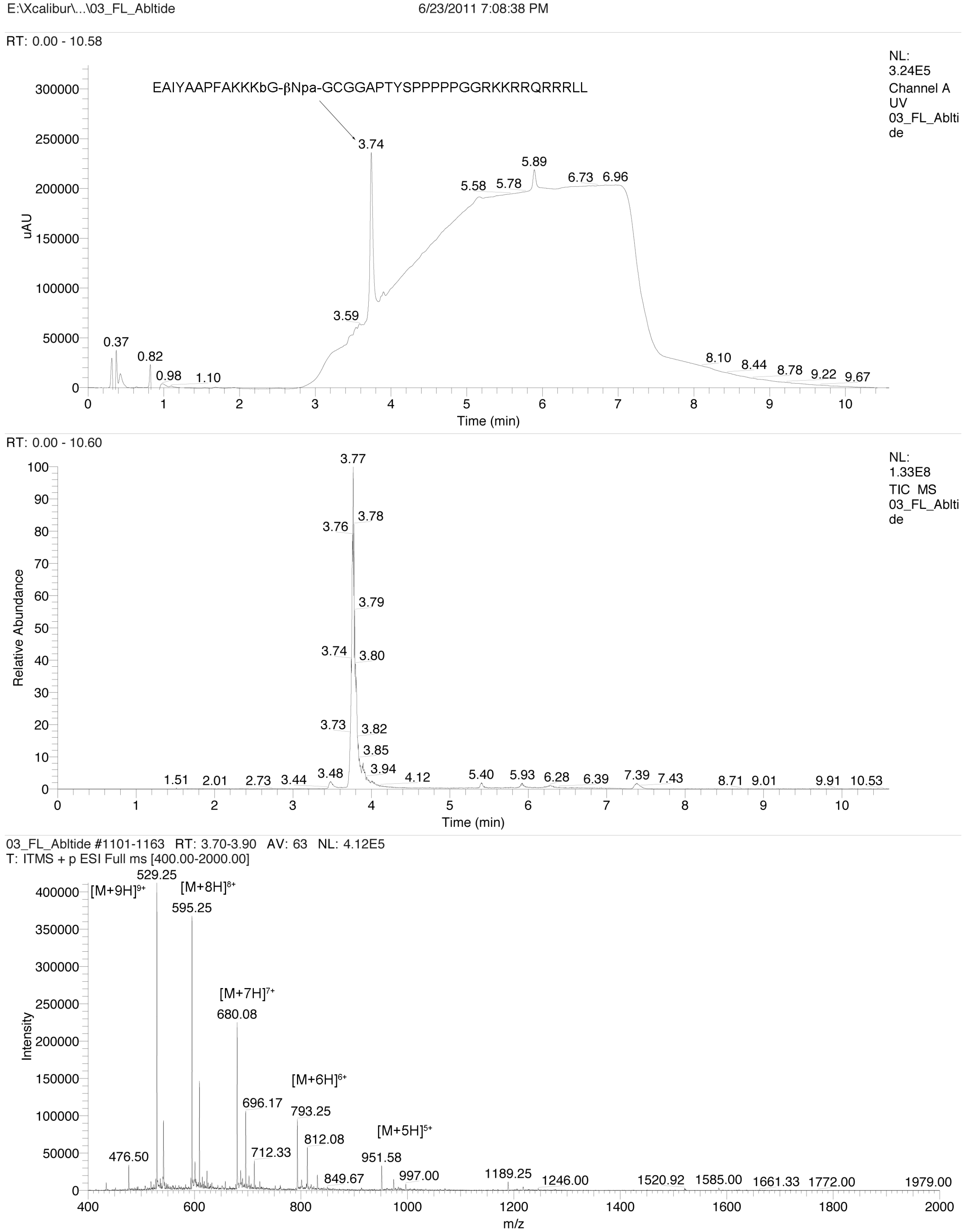

Figure S2. MALDI-TOF characterization of Abl biosensor; shows what appear to be laser-induced photolytic degradation peaks that may represent the intermediates proposed in Scheme S1.[1-2] While the resolution at this mass range makes the m/z differences appear to be 15-17 and 32-34, similar peaks in spectra from other fragments (including Fig. S5 and Fig. S6) show that these losses are more likely to be 16 and 34, and these values are consistent with a plausible proposed mechanism for their formation (Scheme 1). Photolysis peaks are not observed in the LC/ESI-MS spectrum given in S1, indicating that this is a laser desorption mediated process.

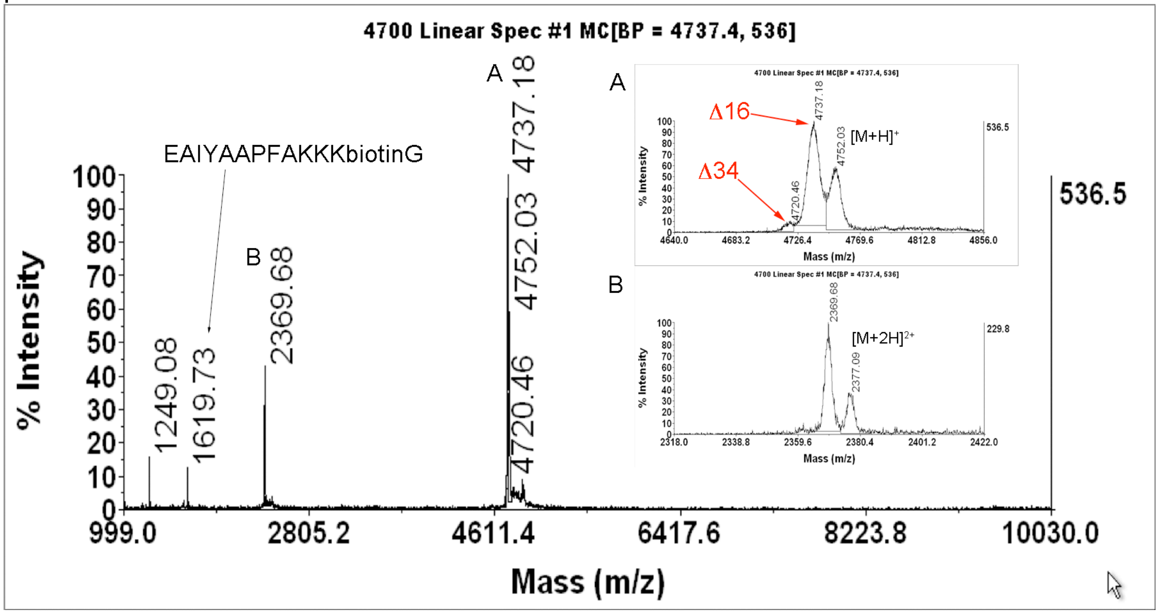

1. Gaplovsky, M.; Il'ichev, Y. V.; Kamdzhilov, Y.; Kombarova, S. V.; Mac, M.; Schworer, M. A.; Wirz, J., Photochemical reaction mechanisms of 2-nitrobenzyl compounds: 2-Nitrobenzyl alcohols form 2-nitroso hydrates by dual proton transfer. *Photoch Photobio Sci* **2005,** *4* (1), 33-42.
2. Il'ichev, Y. V.; Schworer, M. A.; Wirz, J., Photochemical reaction mechanisms of 2-nitrobenzyl compounds: Methyl ethers and caged ATP. *Journal Of The American Chemical Society* **2004,** *126* (14), 4581-4595.

Scheme S1. Proposed potential photodegradation intermediates (as observed in MALDI-TOF spectra). The observed ions exhibit m/z values of 16 amu (major) and 34 amu (minor) less than the expected m/z. The major species is likely formed via gas phase ion chemistry involving protonation of one of the oxygens in the nitro group by initial photo-tautomerization (which is well-characterized in the literature, see 1. and 2. above), additional protonation by acid and elimination as water. For the minor product, the second loss of water (18 amu) to give a total net loss of 34 amu overall could occur—however, the mechanism for this is not clear and while it is very interesting, its absolute determination would be outside the scope of this work. However, this phenomenon suggests that MALDI-MS might be a useful way to study gas phase photochemistry, and this could be the subject of future investigation.

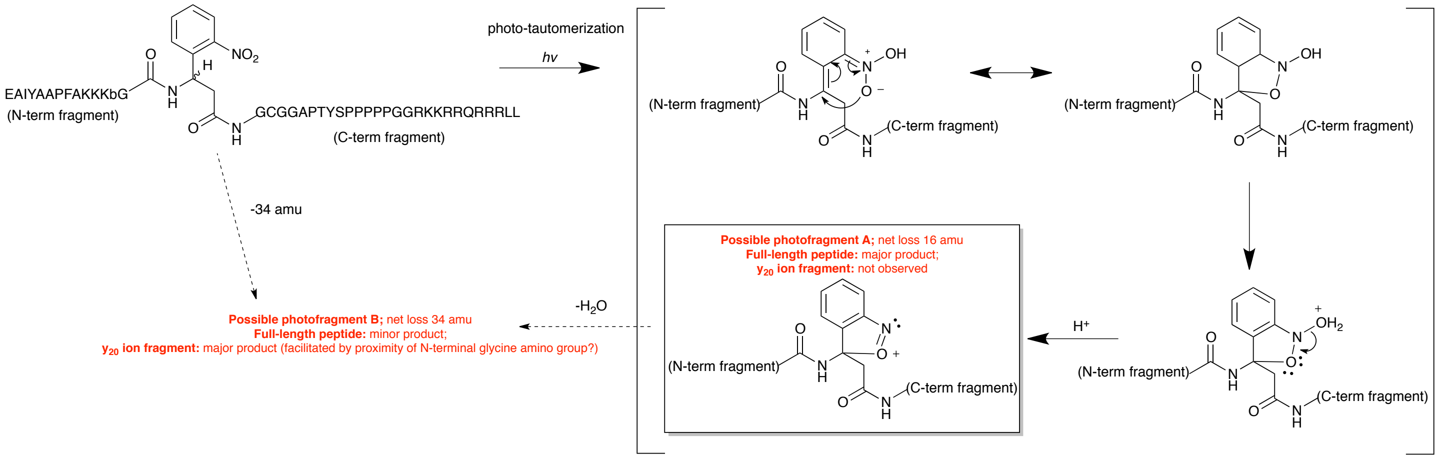

Figure S3. Synthetic phosphorylated Abl biosensor reporter module. Added to K562 lysate followed by digestion and use as MRM calibration standard.

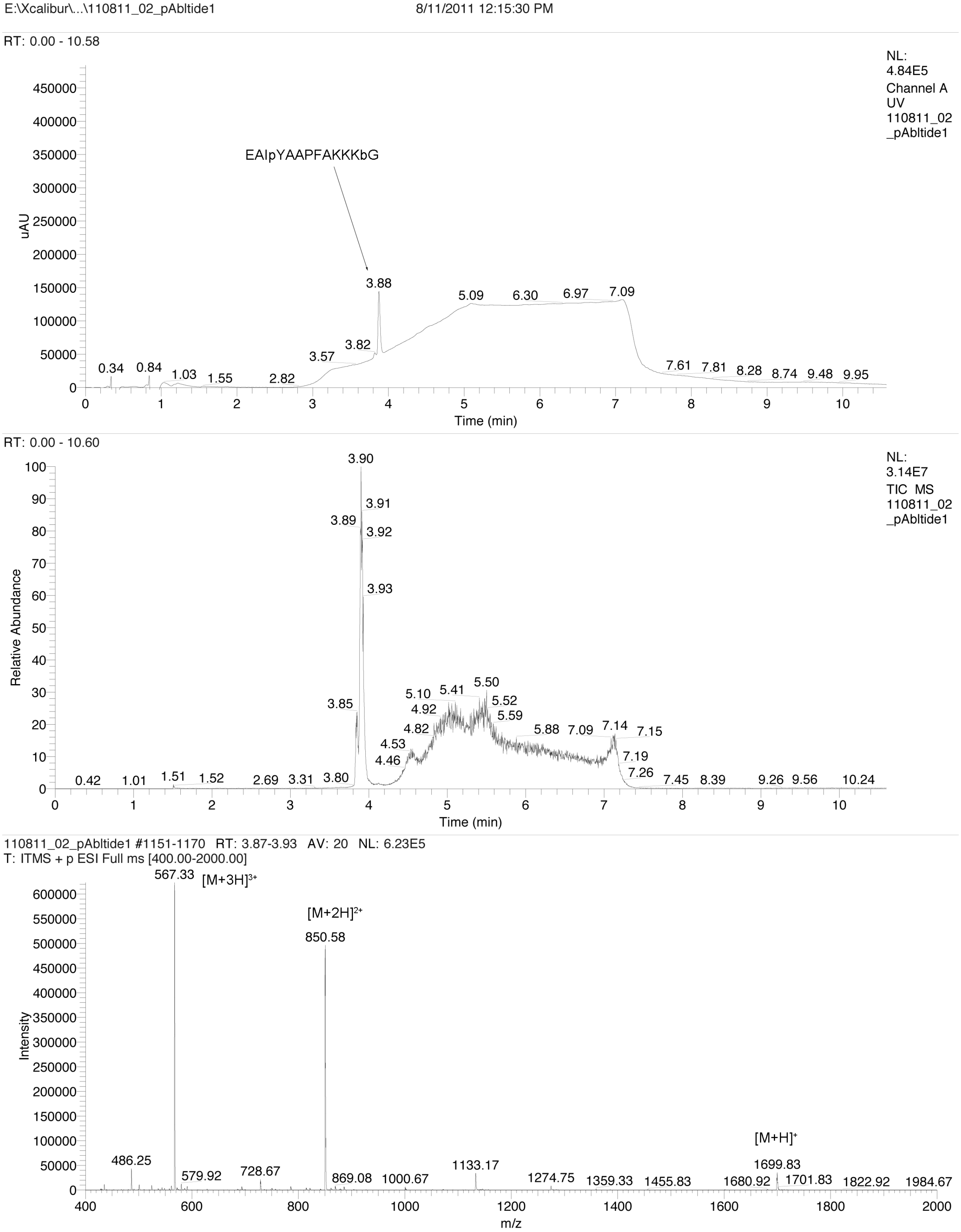

Figure S4. Additional Western blots from all replicates for data shown in Figure 2 in the main manuscript.

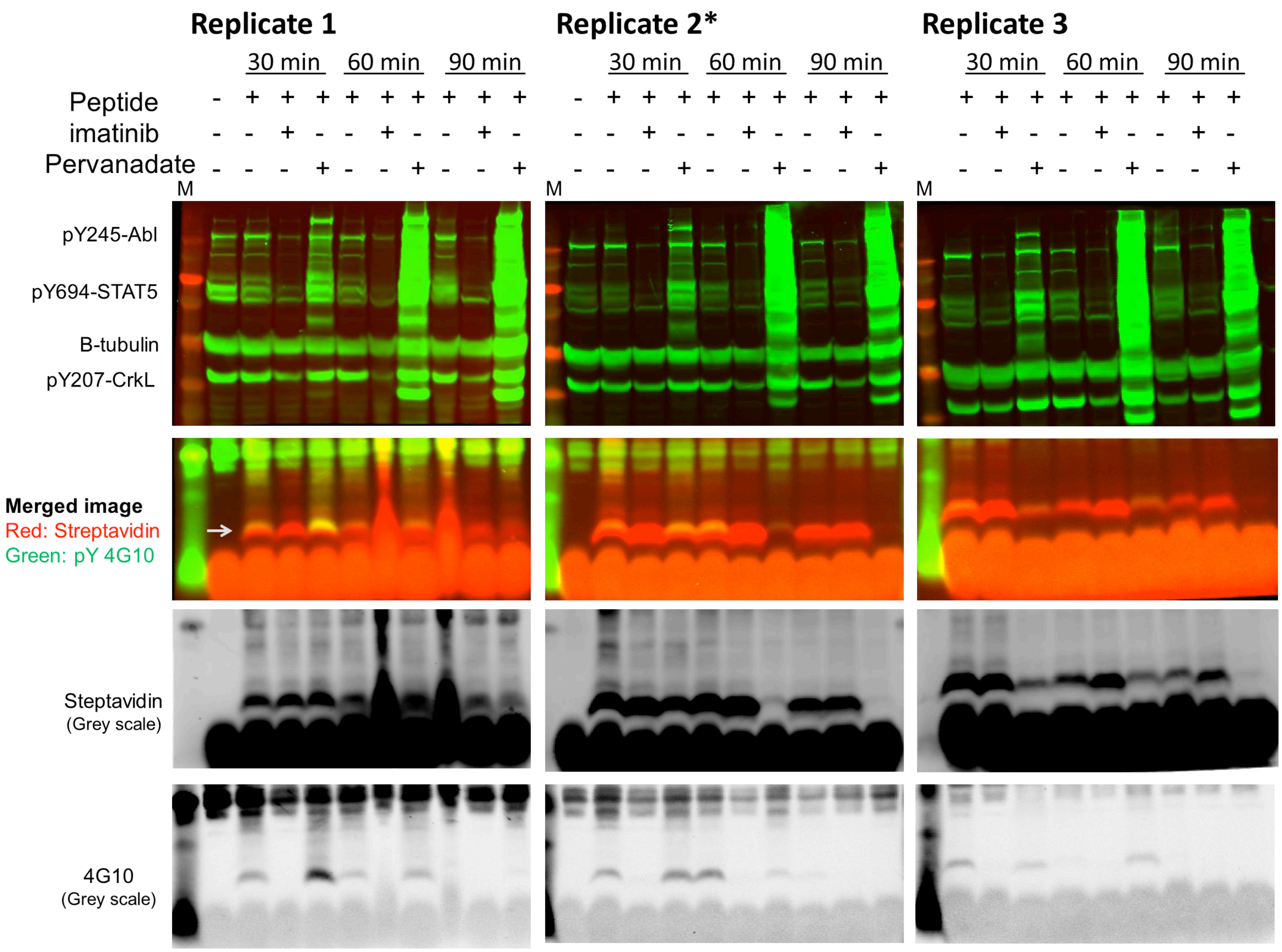

*A grey scale version of replicate 2 was shown in fig 2A.

Figure S5. Expanded view of MALDI-TOF spectrum for degradation products.

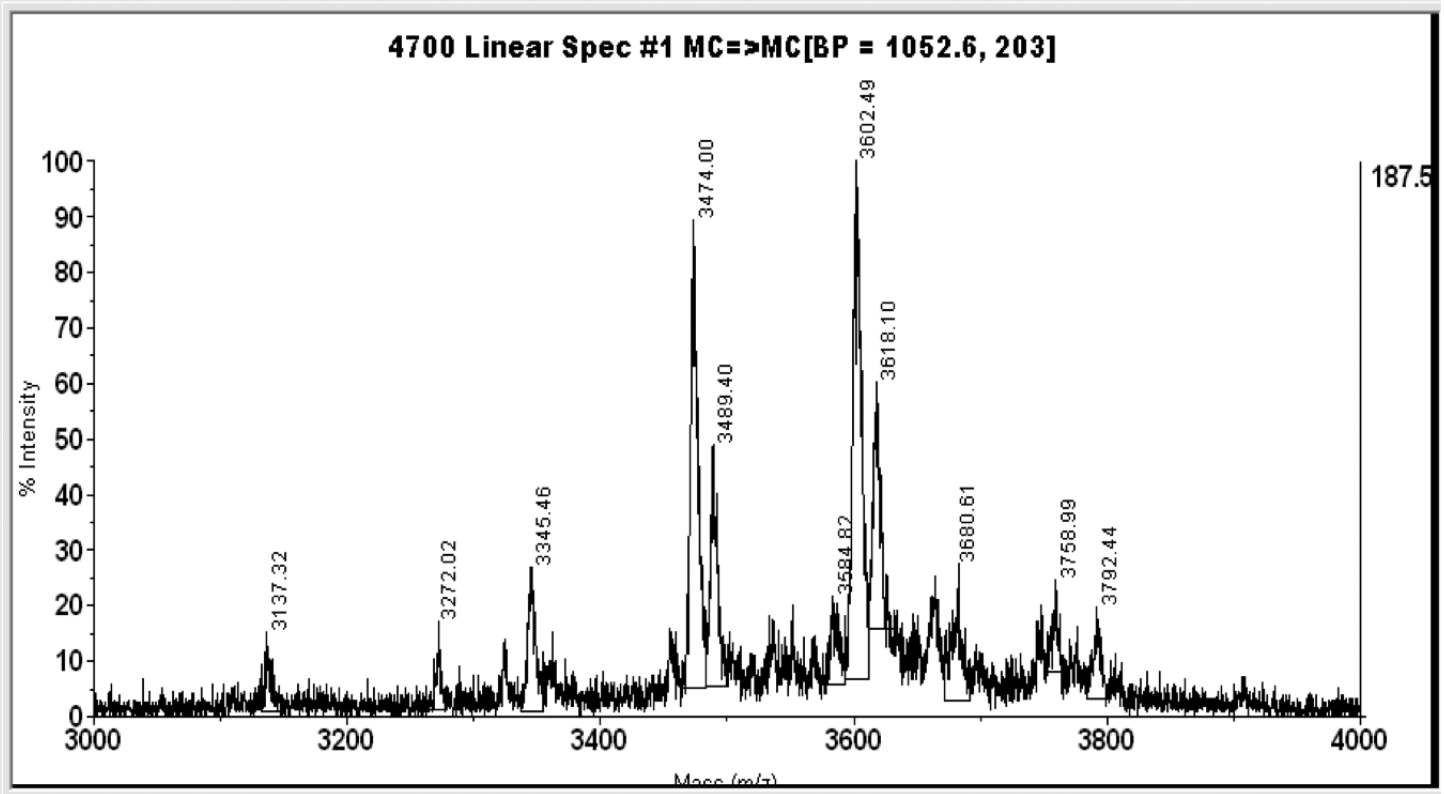

Figure S6. MALDI-TOF/TOF MS/MS for 3618/3602 fragment

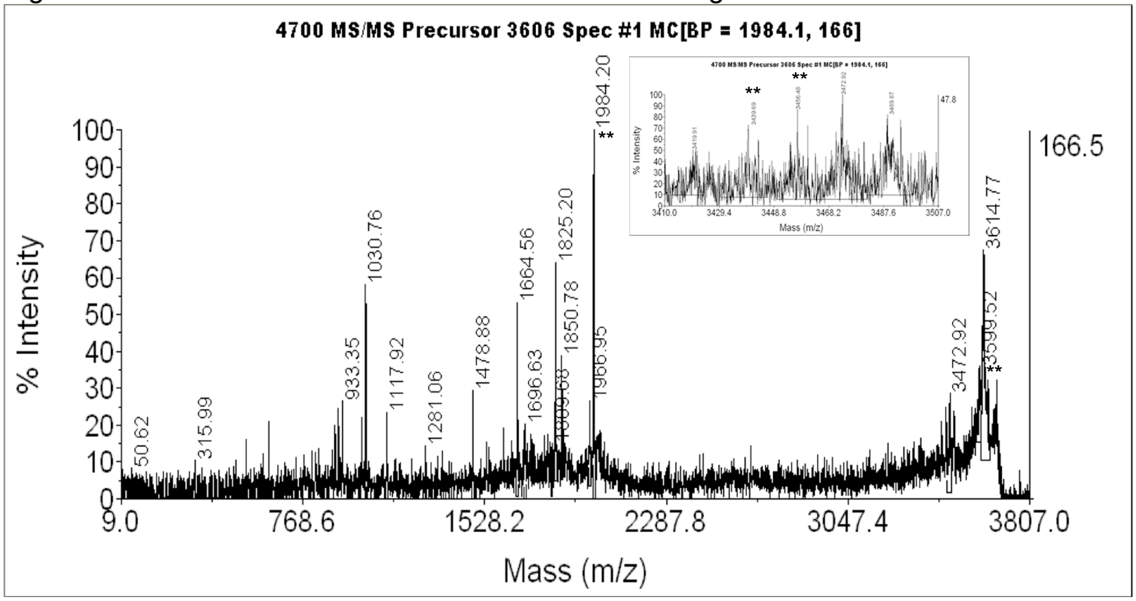

y ion	sequence	m/z	b ion	sequence	m/z
32	EAIYAAPFAKKK$_{biotin}$GβNpaGCGGAPTYSPPPPPGGRKK-OH	3490.96111	1	H2N-E	130.12317
31	AIYAAPFAKKK$_{biotin}$GβNpaGCGGAPTYSPPPPPGGRKK-OH	3419.88251	2	H2N-EA	201.20177
30	IYAAPFAKKK$_{biotin}$GβNpaGCGGAPTYSPPPPPGGRKK-OH	3306.72351	3	H2N-EAI	314.36077
29	YAAPFAKKK$_{biotin}$GβNpaGCGGAPTYSPPPPPGGRKK-OH	3143.54791	4	H2N-EAIY	477.53637
28	AAPFAKKK$_{biotin}$GβNpaGCGGAPTYSPPPPPGGRKK-OH	3072.46931	5	H2N-EAIYA	548.61497
27	APFAKKK$_{biotin}$GβNpaGCGGAPTYSPPPPPGGRKK-OH	3001.39071	6	H2N-EAIYAA	619.69357
26	PFAKKK$_{biotin}$GβNpaGCGGAPTYSPPPPPGGRKK-OH	2904.27431	7	H2N-EAIYAAP	716.80997
25	FAKKK$_{biotin}$GβNpaGCGGAPTYSPPPPPGGRKK-OH	2757.09811	8	H2N-EAIYAAPF	863.98617
24	AKKK$_{biotin}$GβNpaGCGGAPTYSPPPPPGGRKK-OH	2686.01951	9	H2N-EAIYAAPFA	935.06477
23	KKK$_{biotin}$GβNpaGCGGAPTYSPPPPPGGRKK-OH	2557.84591	10	H2N-EAIYAAPFAK	1063.23837
22	KK$_{biotin}$GβNpaGCGGAPTYSPPPPPGGRKK-OH	2429.67231	11	H2N-EAIYAAPFAKK	1191.41197
21	K$_{biotin}$GβNpaGCGGAPTYSPPPPPGGRKK-OH	2075.20231	12	H2N-EAIYAAPFAKKK$_{biotin}$	1545.88197
20	GβNpaGCGGAPTYSPPPPPGGRKK-OH** (observed m/z: 1984.20)	2018.15051	13	H2N-EAIYAAPFAKKK$_{biotin}$G	1602.93377
19	bNpaGCGGAPTYSPPPPPGGRKK-OH	1825.09051	14	H2N-EAIYAAPFAKKK$_{biotin}$GβNpa	1795.99377
18	GCGGAPTYSPPPPPGGRKK-OH	1768.03871	15	H2N-EAIYAAPFAKKK$_{biotin}$GβNpaG	1853.04557
17	CGGAPTYSPPPPPGGRKK-OH	1664.90011	16	H2N-EAIYAAPFAKKK$_{biotin}$GβNpaGC	1956.18417

16	GGAPTYSPPPPPGGRKK-OH	1607.8 4831	17	H2N-EAIYAAPFAKKK$_{biotin}$GβNpaGCG	2013.2 3597
15	GAPTYSPPPPPGGRKK-OH	1550.7 9651	18	H2N-EAIYAAPFAKKK$_{biotin}$GβNpaGCGG	2070.2 8777
14	APTYSPPPPPGGRKK-OH	1479.7 1791	19	H2N-EAIYAAPFAKKK$_{biotin}$GβNpaGCGG A	2141.3 6637
13	PTYSPPPPPGGRKK-OH	1382.6 0151	20	H2N-EAIYAAPFAKKK$_{biotin}$GβNpaGCGG AP	2238.4 8277
12	TYSPPPPPGGRKK-OH	1281.4 9671	21	H2N-EAIYAAPFAKKK$_{biotin}$GβNpaGCGG APT	2339.5 8757
11	YSPPPPPGGRKK-OH	1118.3 2111	22	H2N-EAIYAAPFAKKK$_{biotin}$GβNpaGCGG APTY	2502.7 6317
10	SPPPPPGGRKK-OH	1031.2 4311	23	H2N-EAIYAAPFAKKK$_{biotin}$GβNpaGCGG APTYS	2589.8 4117
9	PPPPPGGRKK-OH	934.12 671	24	H2N-EAIYAAPFAKKK$_{biotin}$GβNpaGCGG APTYSP	2686.9 5757
8	PPPPGGRKK-OH	837.01 031	25	H2N-EAIYAAPFAKKK$_{biotin}$GβNpaGCGG APTYSPP	2784.0 7397
7	PPPGGRKK-OH	739.89 391	26	H2N-EAIYAAPFAKKK$_{biotin}$GβNpaGCGG APTYSPPP	2881.1 9037
6	PPGGRKK-OH	642.77 751	27	H2N-EAIYAAPFAKKK$_{biotin}$GβNpaGCGG APTYSPPPP	2978.3 0677
5	PGGRKK-OH	545.66 111	28	H2N-EAIYAAPFAKKK$_{biotin}$GβNpaGCGG APTYSPPPPP	3075.4 2317
4	GGRKK-OH	488.60 931	29	H2N-EAIYAAPFAKKK$_{biotin}$GβNpaGCGG APTYSPPPPPG	3132.4 7497
3	GRKK-OH	431.55 751	30	H2N-EAIYAAPFAKKK$_{biotin}$GβNpaGCGG APTYSPPPPPGG	3189.5 2677
2	RKK-OH	275.37 051	31	H2N-EAIYAAPFAKKK$_{biotin}$GβNpaGCGG APTYSPPPPPGGR	3345.7 1377
1	KK-OH	147.19 691	32	H2N-EAIYAAPFAKKK$_{biotin}$GβNpaGCGG APTYSPPPPPGGRK	3473.8 8737

Predicted fragmentation for 3618/3602 degradation product
*Observed/tentatively assigned
**Ion observed (m/z 1984.2) has m/z consistent with MS/MS fragment y_{20} exhibiting loss of 34 amu, which may predominate due to some effect of the proximity of the N-terminal glycine in this particular fragment (which, when protonated, could act as a proton donor); other potential photodegradation peaks also marked as ** in spectrum. For further discussion, see Fig. S2 and Scheme S1.

Figure S7. MALDI-TOF to analyze enriched reporter segment.
Replicate 1:

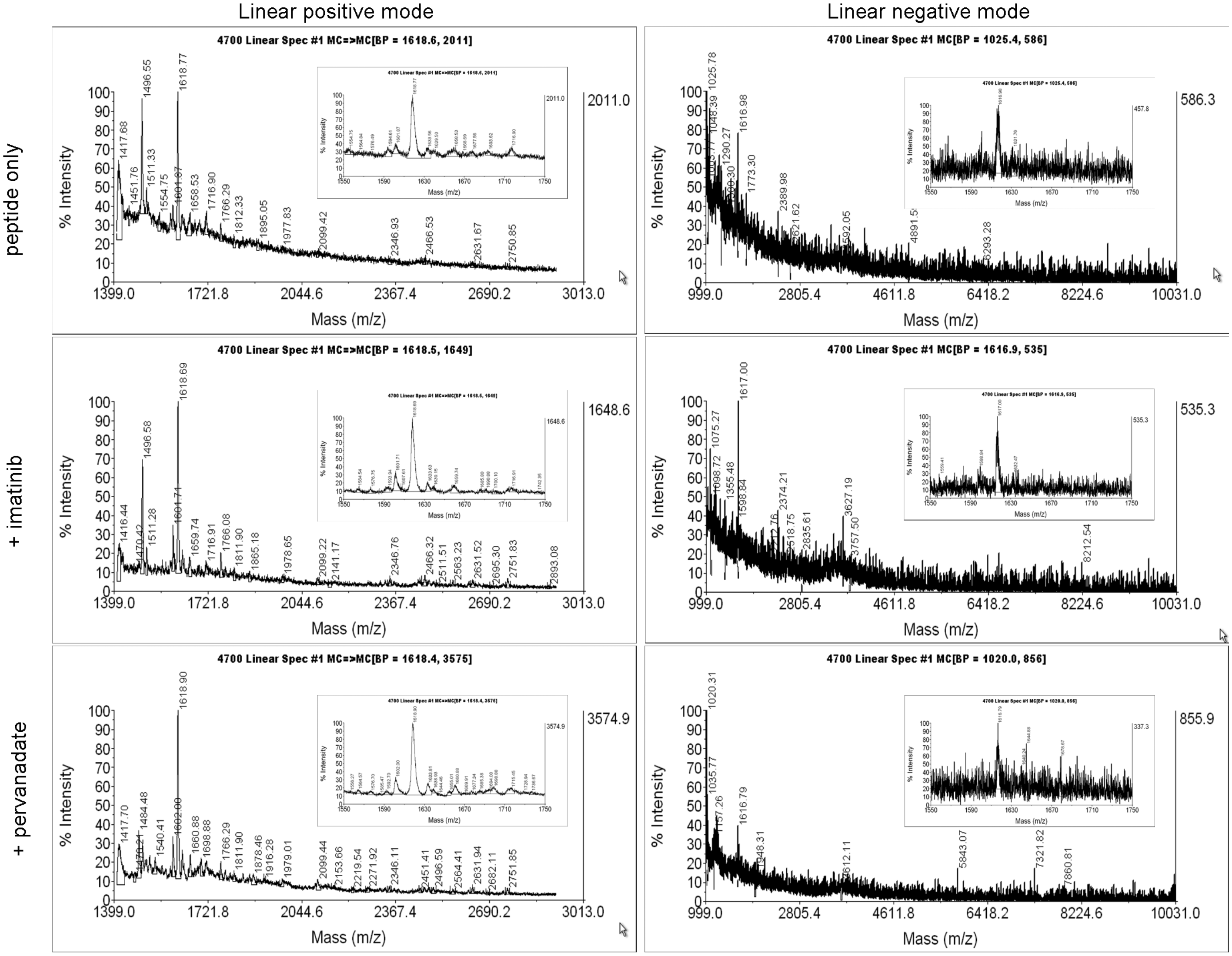

Replicate 2:

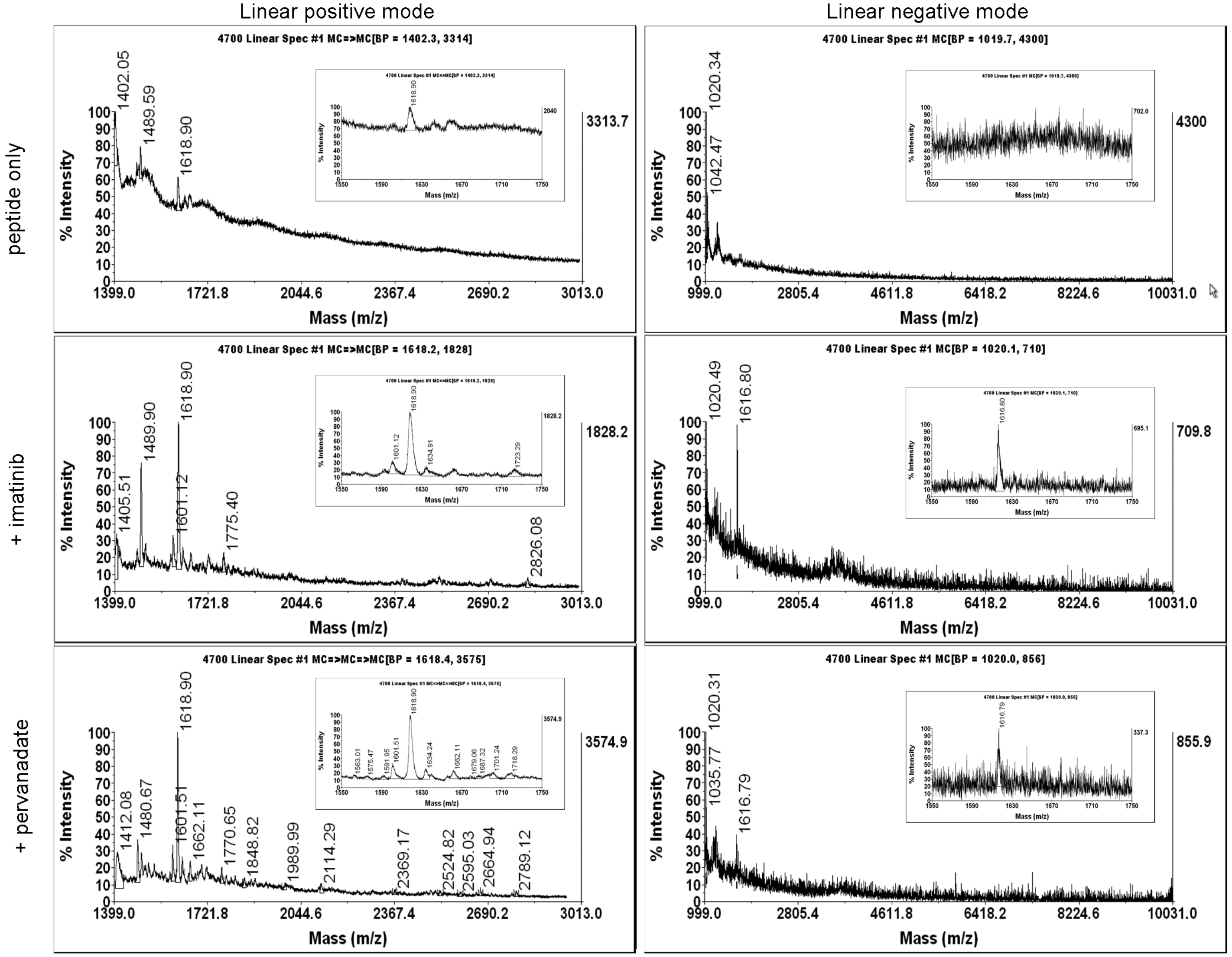

Table S8. Table of raw MRM data. See additional .csv file.

Table S9. Table of peak integrations and calibration. See attached Excel file.
